# 3D-printed Laponite/Alginate hydrogel-based suppositories for versatile drug loading and release

**DOI:** 10.1007/s13346-023-01506-5

**Published:** 2024-01-07

**Authors:** Elena Munoz-Perez, J. Rubio-Retama, Lorena Cussó, Manoli Igartua, Rosa Maria Hernandez, Edorta Santos-Vizcaino

**Affiliations:** 1 NanoBioCel Research Group, Laboratory of Pharmaceutics, School of Pharmacy, University of the Basque Country (UPV/EHU), Paseo de la Universidad 7, 01006 Vitoria Gasteiz, Spain; 2 NanoBioCel Research Group, Vitoria Gasteiz, Spain; 3 Department of Chemistry in Pharmaceutical Science, Complutense University of Madrid, 28040 Madrid, Spain; 4 Unidad de Imagen Avanzada, Centro Nacional de Investigaciones Cardiovasculares Carlos III (CNIC), Madrid, Spain; 5 Laboratorio de imagen para pequeño animal de experimentación, Instituto de Investigación Sanitaria Gregorio Marañón, Madrid, Spain; 6 CIBER de salud mental, Instituto de salud Carlos III, Madrid, Spain; 7 Biomedical Research Networking Centre in Bioengineering, Biomaterials and Nanomedicine (CIBER-BBN), Institute of Health Carlos III, Madrid, Spain

**Keywords:** 3D printing, Semi-solid extrusion, Laponite, Alginate, Drug-delivery

## Abstract

Traditional approaches to solid rectal therapies have halted progress, leading to a continual decline in the use of conventional suppositories. Additive manufacturing techniques have been recently explored as a suitable innovative tool for suppository fabrication. However, little advancement has been made in composition materials for 3D-printed suppository (3DPS) manufacturing and still, conventional vehicles are often used for construct fabrication, hindering the growth in the field. As a novelty, this study unveils a ground-breaking Laponite-alginate hydrogel-based 3DPS. Interestingly, this study proposes a novel approach for loading drugs into the 3DPS employing for the first time the post-printing loading. Thus, a passive loading strategy of molecular models is developed, demonstrating the versatility and capacity to load molecules of different charges and molecular sizes within the matrix systems. This novel strategy allows adapting the load of a wide range of drugs into a single ink, which simplifies and speeds up the 3DPS technological development process for drugs with different physico-chemical properties. Additionally, in this research, a displacement strategy of the three-dimensional Laponite matrices is developed in order to enhance the drug release capacity through the 3DPS and their disintegration capacity, resulting in a significant improvement of the drug diffusion through the hydrogel matrix and a rapid disintegration of the 3DPS. Finally, our study demonstrates that the obtained 3DPS have a suitable in vivo behavior, being non-obstructive and allowing the normal motility of the rats intestine.

**Graphical abstract:**

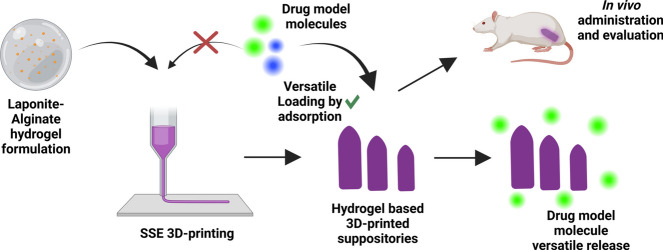

## Introduction

Solid rectal therapies are witnessing a decline in usage and a great responsible for this downward trend can be attributed to a lack of progress in their production methods [1, 2]. The most commonly employed manufacturing method of suppositories is the moulding method and it presents significant constraints towards the innovation of novel solid rectal therapies. Among others, moulding manufacturing limits the size and shape of formulations to the available manufacturing moulds, hampering the customization of treatments [3]. Thus, dosage of the conventionally manufactured treatments has proven to be poorly adaptable to the individual needs of patients [4]. Moreover, the variety of commercial doses for suppository-based treatments is limited, necessitating the use of individual pharmaceutical compounding methods for therapy personalization [5]. Compounding methods for suppository manufacturing escalate the healthcare expenses for patients since they require a skilled labour for their development and additional material costs. Besides, they are usually related to low yield production capacity. Furthermore, inaccuracies in moulding methods often occur due to the shrinking or expanding effects on the formulation mixture caused by the heating and cooling involved. Consequently, there is a prevalence of underdosing or overdosing in patients treated with compounding-manufactured suppositories, usually related to technical difficulties involved in the manufacturing processes. On the contrary, 3D printing (3DP) techniques can employ not fully melted mixtures during the formation of the dosage form, thereby avoiding inaccuracies in the manufacturing process [6].

Therefore, novel approaches are imperative for the personalized production of suppositories, which allow a clear innovation in the field and provide versatility to the formulations. Additive manufacturing arises as a leap forward in 3DPS production for several reasons. Firstly, 3D printing techniques empower the tailoring of shape, size, and dosage of therapies [1–9]. More specifically through digital editing of the 3D design, alterations can be made in dosage forms without necessitating extra expenses or the time typically required for additional moulding in fusion processes. Similarly, additive manufacturing offers the possibility to adapt personalized formulation batches sizes to the patient’s needs [10]. Moreover, it is a straightforward technique that entails no difficulty once set up, minimizing complexities and reducing human intervention [11]. This not only helps to prevent errors, but also reduces the need for highly skilled labour [3]. All these advancements can alleviate the healthcare and material costs involved in conventional 3DPS fabrication methods and position 3D printing as a novel benchmark.

The most widely used 3D printing technique for manufacturing novel 3D-printed suppositories (3DPS) is the semi-solid extrusion 3D printing (SSE-3DP] of melted materials [3, 8, 12, 13]. This approach utilizes melting pastes, waxes or gels of different materials, such as Polyvinyl alcohol (PVA), lipids, and poly-ethylene glycol (PEG), which can solidify and acquire the desired shape at room temperature following the extrusion process [14, 15]. This 3D printing technique provides the capability to print customized pharmaceutical shapes in various sizes and forms without requiring extremely high temperatures that might compromise non-thermostable drugs. Furthermore, this technique presents a clean and straightforward alternative for 3D printing pharmaceutical shapes [8]. However, the use of this 3D printing technique has some limitations related to resolution and reproducibility of the printlets [16, 17]. For example, inaccuracies related either to the 3D-printing parameters — nozzle diameter and printing settings, or the printer employed for the process might led to temperature variations during printing procedure. These parameters can also affect post-printing hardening processes, led to variations in printlets shape and affect negatively the printing accuracy decreasing the reproducibility [18]. Additionally, preserving the integrity of the obtained 3D printed formulations poses challenges, particularly in terms of refrigeration requirements. This way, it is worth noting that while SSE-3DP offers advantages, the current application of this technique with melted materials still grapples with the need for refrigeration for printed rectal pharmaceutical forms [19].

Thus, there is an unmet need to implement novel materials in the additive manufacturing of 3DPS that can overcome the existing limitations described above. In this regard, extrusion-based 3D printing of non-melted hydrogels emerges as a promising alternative for this aim. Unlike melting materials extrusion for SSE-3P, hydrogel extrusion process is only limited by the flow characteristics of the hydrogel ink employed for 3D printing [9]. Thus, shear-thinning capacity of hydrogel inks is not usually affected by temperature changes, ensuring a high level of control over variations in the flow throughout the entire printing process. Likewise, conservation of hydrogel based biomedical devices is simple and does not usually require refrigerated conditions. Additionally, tunability in hydrogel formulation enables novel material compositions to be applied in suppository 3D printing [20–22]. Furthermore, hydrogels have gained wide acceptance for biomedical applications, due to their demonstrated biocompatibility, high water content, softness, ease of formulation and manipulation, as well as non-irritating properties [23].

As a promising hydrogel composition, the combination of a highly biocompatible polymer such as alginate (Alg) with Laponite (Lap) nanoclay has been shown to ensure easy extrudable inks [24]. Thus, in our previous studies, we optimized the development of Lap/Alg hydrogel-based ink as well as its printing parameters for its employment in SSE-3P [9]. Lap/Alg inks demonstrated to have outstanding flow properties, printing accuracy and stackability, as well as being highly biocompatible [9]. Moreover, different studies have shown that Lap/Alg composite systems offer characteristics of great interest for drug delivery applications [25, 26]. The three-dimensional assembly structure of the nanoclay has demonstrated to have a high capacity for drug adsorption [27]. Besides, Lap has been associated with sustained drug delivery [25]. This remarkable behavior can be attributed to the extensive surface area of Lap plates, which facilitates efficient adsorption of molecules through strong ionic interactions with drug molecules. On the other hand, alginate offers the possibility of applying ionic crosslinking to Lap assembly, allowing the formation of robust three-dimensionally hydrogel systems with enhanced mechanical resilience, water retention and stability [9].

However, despite the fact that 3D printing biomedical devices with Lap/Alg hydrogels offers good results and promising features, the use of Lap/Alg hydrogel-based 3D printed biomedical devices as drug delivery systems has never been widely exploited. This is likely because the robust drug-Lap interactions have proven to be difficult to displace, conferring high drug adsorption capacity but constraining drug release from 3D printed Lap/Alg systems [28]. These limitations hinder the effective dosing of treatments and impede the widespread use of Lap/Alg hydrogels as reliable drug delivery systems. In this study, we developed a novel Lap/Alg hydrogel-based 3DPS and addressed the common limitations of Lap/Alg-based drug delivery systems. Thus, this study first proposes the technological development of the Lap/Alg hydrogel ink based 3DPS. We analyze the most efficient way of printing the system as well as its printability and morphology. Likewise, this study proposes a disintegration strategy to increase the disintegration capacity of the Lap/Alg system. In this way, the use of BSA (Bovine Serum Albumin) as an ionic displacement strategy to increase the disintegration of the system is analyzed. Furthermore, this study proposes 3DPS as versatile loading pharmaceutical forms, proposing the adsorption loading strategy as a versatile drug loading mechanism for the system and analysing the mechanism underlying the high loading capacity. Similarly, this development proposes an ionic displacement mechanism as an enhancer of drug release from the system, addressing the usual limitations in the use of Lap in drug delivery systems. Accurately, we analyzed the adsorption loading capacity and release ability of drug model molecules of different molecular weights and charges into the 3D printed system. Finally, as an unprecedented approach for Lap/Alg hydrogels, we completed our research with an in vivo administration of the 3DPS and an analysis of its rectal occlusion capacity and over time behavior inside rats colon.

## Materials and methods

### Hydrogel ink preparation

Hydrogel ink was prepared as described in previous studies [9]. Briefly, Lap was dispersed with gentle stirring in deionized water at a concentration of 6% (w/v). Then, sodium alginate was added and dispersed into Lap dispersion reaching a final concentration of 1% (w/v). For a better alginate dispersion, an ultra-turrax system was employed for dispersion until no aggregates were observed in the final hydrogel. The formed hydrogel ink was transferred into 3 mL Luer-lock syringes and centrifuged at 3500 rpm for up to 15 min to remove bubbles.

### 3DPS 3D models design

The software *AutoCAD* was employed to design the 3D models of suppositories. Three different sizes of models were developed ranging from the small (6 mm height × 3 mm width; divided in 1.5 mm radius sphere and a 4.5 mm cylinder), medium (8 × 4 mm; divided in 2 mm radius sphere and a 6 mm cylinder) and large (12 × 10 mm; divided in 5 mm radius sphere and a 7 mm cylinder) sizes. Once the printability of the ink was tested, small size 3DPS were employed for the rest of the study.

### Semi-solid extrusion 3D printing process

The hydrogel ink was transferred into a 3 mL printing cartridge in order to be printed. Thus, 22 G printing conical nozzles and 35 kPa extrusion pressure were employed for printing. Designs were printed into 24 well-plates and cross-linked using a CaCl2 bath for 10 min.

### Optimization of 3DPS drug-loading strategy

In order to determine the most efficient strategy for drug-loading the 3DPS, two different strategies were compared: Loading the Lap/Alg ink with the actives prior to printing and loading the 3DPS once printed and cross-linked by means of a passive adsorption strategy.

The influence of both loading strategies was tested and compared in terms of printability of the loaded and blank inks (Table 1). Then, the drug loading capacity of non-loaded 3DPS was tested by means of passive loading adsorption of methylene blue into 3D-printed non-loaded suppositories.

**Table 1:** Methylene blue loaded and unloaded inks detailed composition

**Ink name**	**Alginate % (w/v)**	**Laponite % (w/v)**	**Methylene blue %(w/v)**	**Water content (%)**
**Methylene blue loaded ink**	1	6	10	83
**Blank Lap/Alg ink**	1	6	0	93

#### Influence of drug loading strategy on 3DPS printability

In order to test the influence of loading the ink prior to the extrusion process, 1 mL of ink was loaded with a 10% (w/v) methylene blue solution by means of the double syringe mixing method. The loaded ink was then transferred into a 3 mL printing cartridge and printed employing 22 G printing conical nozzles and 35 kPa extrusion pressure (Table 1). Similarly, the blank Lap/Alg inks (Table 1) were also printed employing the same parameters. The deflection ratio and printing accuracy of both types of inks were determined and compared as described below.

##### Deflection ratio

The area of the 3DPS was calculated in relation to the computer-designed area (70.6 mm^2^) (Eq. (1)). A ratio closer to 100 indicates a higher similarity between the printed and designed areas.

1 $$Deflection\;Ratio =\frac{Printed\;area}{Design\;area} \times 100$$

##### Printing accuracy

The area of the printed and cross-linked 3DPS was compared to the computer-designed area (Eq. (2)). Results close to 100 were attributed to a higher printing accuracy in the process.

2 $$Printing\;Accuracy =\left[1-\frac{\left(Printed\;area-Design\;area\right)}{Design\;area}\right] \times 100$$

#### Drug loading capacity of non-loaded 3DPS

The loading capacity through passive adsorption of the non-loaded 3DPS was tested. Briefly, formulations were printed, cross-linked and immersed in a bath of 1 mL of 1% (w/v) methylene blue solution for 24 h. After this time, the adsorption of methylene blue inside the formulation was observed by indirect measurement of the remaining concentration in the bath solution and visual analysis of the formulation. The absorbance of methylene blue was measured at both time 0 and 24 h by means of spectrophotometry (Infinite® 200 PRO series, Tecan Trading AG). A 656 nm wavelength was selected as the maximum absorbance for the analysis of Methylene Blue. The absorbance values obtained were then correlated to the concentration of methylene blue using a calibration curve generated with known standards. The difference between the initial concentration of methylene blue and its final concentration in the adsorption solution was considered to be attributable to its adsorption inside the 3DPS.

Photographs of the 3DPS immersed in methylene blue dissolution at time points 0 and 24 h were taken in order to visualize differences.

The results of the experiments carried out up to this point in the development were considered sufficient to discard loaded-ink printed 3DPS as efficient for drug loading. Therefore, the rest of the studies were carried out with formulations printed with blank Lap/Alg ink.

### Cryo-SEM analysis of printed 3DPS

Horizontal SEM images of the 3DPS were taken by means of table-top TM4000Plus SEM (Hitachi) equipped with a coolstage (Deben UK Ltd). Cryo-SEM analysis was performed at 5 kV using a backscattered electron detector/secondary electron detector.

### Employment of BSA displacement strategy in 3DPS disintegration, drug-adsorption and drug-release stages

**Fig. 1:**
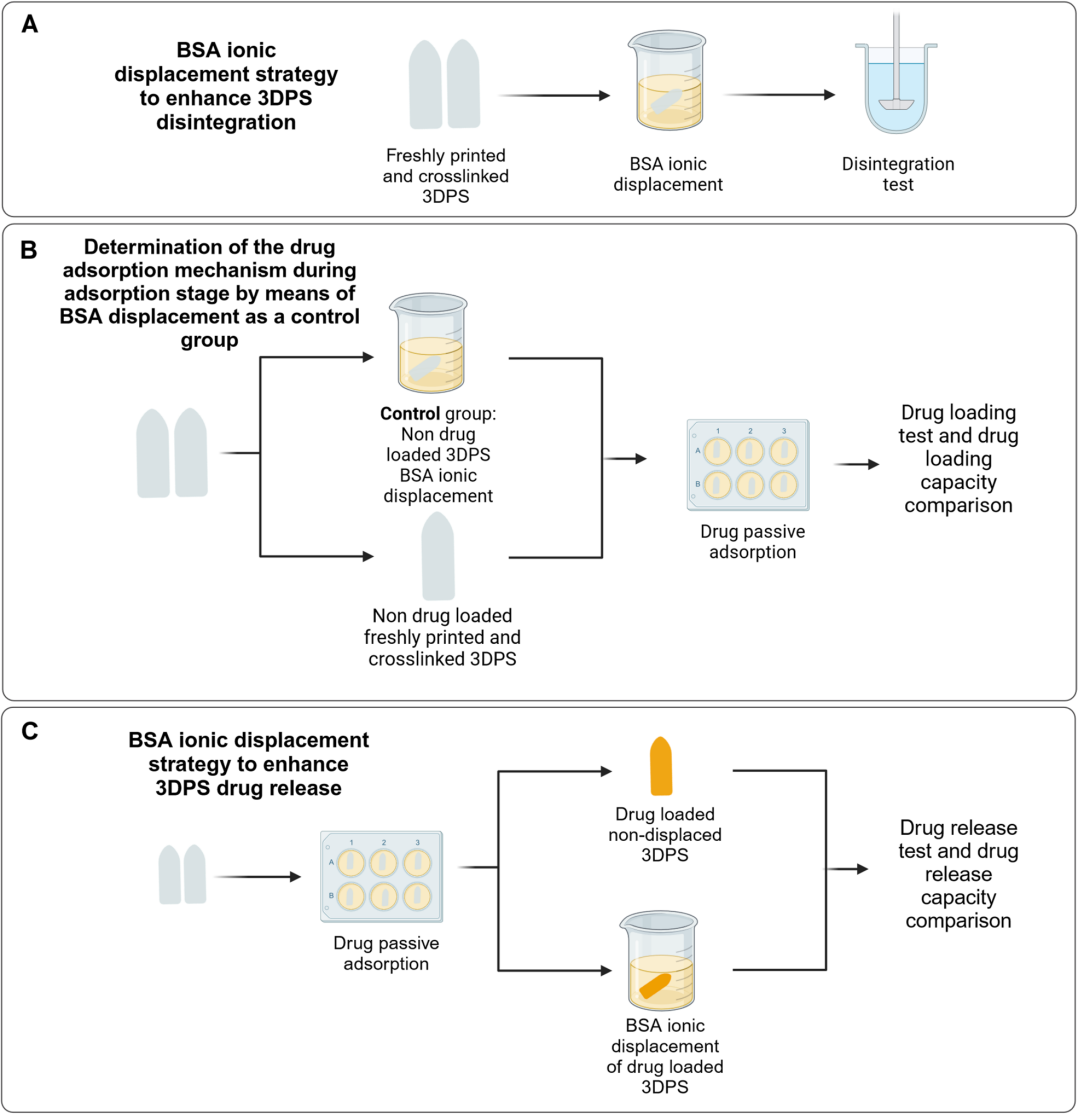
BSA displacement strategy employed in disintegration, drug-loading and drug-release of 3DP-SRF. **A** BSA displacement strategy employed as a disintegration enhancer in 3DPS disintegration test. **B** BSA displacement strategy employed as a control group during the adsorption tests of 3DP-SRF in order to clarify the underlying adsorption mechanism **C** BSA displacement strategy as a drug release enhancer for 3DPS

To promote 3DPS disintegration, BSA was employed as an ionic displacer. Thus, prior to 3DPS disintegration, freshly printed and cross-linked formulations were immersed in BSA solution for enhancing their disintegration capacity (Fig. 1A). The study was used to optimize the concentration and exposure time to BSA that elicited the most rapid disintegration.

The influence of the BSA ionic displacement strategy in the drug loading capacity of the 3DPS was also determined by comparing the adsorption capacity of displaced — BSA bath immersed — and non-displaced 3DPS (Fig. 1B). The ionic displacement strategy using BSA during the adsorption stage was not performed as a drug adsorption control strategy for the formulations. Instead, during the loading stage, displacement prior to adsorption was used to determine the underlying mechanism of drug loading into the 3DPS. Thus, the formulations displaced before the adsorption stage were used as a control group to inhibit the adsorption mechanism of the Laponite plates and assign the drug loading capacity of the displaced formulations to a mere passive diffusion mechanism.

Contrarily, the BSA ionic displacement strategy was also employed to enhance 3DPS drug release capacity. Therefore, once the passive drug loading of 3DPS was performed, the formulations were immersed in a BSA solution for enhancing their release capacity. Then, drug release test was performed. Displaced and non-displaced formulations were compared within all of the preformed drug release tests (Fig. 1C).

### 3DPS BSA displacement optimization for formulation disintegration

For controlling formulation disintegration time, the BSA ionic displacement strategy was optimized. Thus, BSA was dissolved in 50 mL of phosphate saline buffer (PBS) at concentrations of 5, 10 and 15% (w/v). Freshly printed and cross-linked formulations were immersed in 1 mL of displacement solution for 30 min, 1 h, 2 h, 3 h or 4 h. Influence of the displacement strategy in 3DPS disintegration was tested employing a disintegration apparatus slightly modified to match our requirements (USP I SOTAX AT 7). Briefly, a disintegration apparatus was tuned with filter cages of 1 mm pore size where 3DPS were placed and observed until disintegration. 1 L of water was employed as disintegration media and a frequency of 10 rpm. Cages were opened for formulation state determination in times 10, 20, 40, 90 and 120 min. Formulation disintegration time was established in time ranges defined as: (S1) 10–20 min, (S2) 20–40 min, (S3) 40–90 min, and (S4) ≥ 120 min. The absence of formulation debris when opening the cages was assumed to be due to disintegration. Non-displaced formulations were employed as non-disintegration controls.

Similarly, disintegration time of freshly printed, cross-linked and displaced 3DP2 was tested under more restrictive conditions. Thus, 3DPS were placed in a 15 mL tube containing 1.5 mL of PBS under gentle orbital shaking conditions — 20 rpm. Formulation disintegration time was established in time ranges defined as: (S1) ≤ 4 h, (S2) 4–6 h, (S3) 6–8 h, and (S4) ≥ 8 h. Non-displaced formulations were employed as a non-disintegration control. Each displacement time and concentration were tested in triplicate for both disintegration conditions.

### 3DPS passive adsorption variability determination

Adsorption capacity of freshly printed and cross-linked 3DPS was determined. Thus, freshly printed and cross-linked 3DPS were immersed in 1 mL of 0.5 mg/mL Fluorescein-isothiocianate-dextran (FITC-dextran) (Sigma Aldrich ®) PBS solution for 24 h. A total volume of 200 uL of sample was withdrawn and replaced with fresh PBS media on time points 1, 2, 4, 8 and 24 h. Fluorescence intensity of samples was determined by means of a fluorescence plate reader (Infinite® 200 PRO series, Tecan Trading AG). In order to test the influence of the molecular size on 3DPS adsorption, the experiment was performed with FITC-dextrans of 10, 40, 70 and 150 kDa. Similarly, the influence of molecular charge on the adsorption was determined employing negatively charged FITC-carboxymethyl-Dextran (FITC-CM-DEXTRAN Sigma Aldrich®) and positively charged FITC-Diethylaminoethyl-Dextran (FITC-DEAE-DEXTRAN Sigma Aldrich®). In order to test the influence of the BSA displacement strategy during 3DPS adsorption stages, a BSA-displaced group was included in the assay for each of the FITC dextran tested. Thus, printed and cross-linked 3DPS were immersed in a BSA 15% solution for 1 h prior to adsorption assay. Finally, we calculated the area under the curve (AUC) for the different types of dextrans in displaced and non-displaced formulations. This allowed us to compare the influence of the displacement strategy on the adsorption capacity of the 3DPS and to clarify adsorption mechanisms involved.

### 3DPS Adsorption layer depth

To identify the location of the adsorbed dextran molecules after the adsorption study, the thickness of the dextrans adsorption layer in the 3DPSs was determined. For this, the 3DPSs were cut transversely into 1 mm thick slices. The external part of the slices was observed and photographed in a fluorescence microscope (Nikon) equipped with a DSD2 confocal modulus. Slices of three different 3DPS were observed for each of the loaded dextran. The dextran adsorption layer depth was measured and compared using Image J software (NIH).

### 3DPS release capacity measurement

Previously loaded 3DPS were used for measuring their dextran-release capacity immediately after loading. Thus, 3DPS loaded with FITC-dextrans of 10, 40, 70, 150 kDa, 40 kDa positively charged (FITC-DEAE-dextran) or 40 kDa negatively (FITC-CM-Dextran) charged were employed for the study. 3DPS were placed in a 1.5 mL PBS loaded 15 mL tube under gentle orbital shaking at 20 × rpm by means of an orbital shaker. A total volume of 200 uL of sample was withdrawn and replaced with fresh PBS media at time points 1, 2, and 4 h. Samples were centrifuged (4000 × rpm) to pellet 3DPS residues and fluorescence intensity of supernatants was determined employing a plate reader. To test the influence of the BSA during the 3DPS release stage, a BSA-displaced — 15% (w/v) during 1 h — group was included in the assay for each of the FITC dextran tested. The dextran release capacity of displaced and non-displaced formulations was compared. Finally, we calculated the AUC for the different types of dextrans in displaced and non-displaced formulations. This allowed us to compare the influence of the displacement strategy on the release capacity of the 3DPS. We compared the AUC for each dextran type in both the displaced and non-displaced formulations.

### In vivo administration of 3DPS

12 Wistar Han male rats with an average weight of 200 g were used for the study. Animals were fasted for 12 h before the study with free access to drinking water. The formulations were pre-stained with 10 mg of a CT contrast agent based on pegylated Au nanoparticles and inserted into the rectum of the animals with the help of forceps — 4 cm distal to the anus. The anus of the animals was sealed by applying sutures. The administration of the formulation, anal sealing as well as CT image acquisition were performed with the animal anesthetized (3% sevoflurane in 100% oxygen). CT studies were carried out with a SuperArgus small-animal scanner (SEDECAL, Madrid) with an X-ray beam current of 340 μA and a tube voltage of 40 kVp. We reconstructed the images with the Feldkamp, Davis, and Kres (FDK) algorithm [29]. CT were acquired at time points 0, 1, 2, 3, 4 and 6 h after administration and animals were allowed to recover from anesthesia after each acquisition. After the last image, the animals were euthanized and the contents of the rectum were extracted to observe the state of the formulations.

The images were analyzed with the Multimodality Workstation tool (MMWKS, Spain) [30]. A region of interest (ROI) was drawn from over the entire volume of the formulation at each time point to obtain the volume, total brightness and average brightness/volume.

In order to evaluate the influence of the displacement strategy in 3DPS performance in vivo, animals were divided into two different groups described as: Non-displaced 3DPS administered group (*n* = 6) and Displaced 3DPS administered Group (*n* = 6).

### Statistical analysis

One-way ANOVA was employed to analyze normally distributed results. Bonferroni or Tamhane post hoc analysis was applied founded on the homogeneity of variances Levene’s test. Non-normally distributed values were analyzed by Mann–Whitney analysis. Results were expressed as the mean ± SD. Statistical analysis was performed using SPSS 25.0 (SPSS®) considering *p* < 0.05 as statistically significant. The sample sizes for each experiment are displayed in figure captions.

### Ethics

Mice were housed in the animal facility of the Hospital General Universitario Gregorio Marañón, Madrid (HGUGM), Spain (ES280790000087). All animal procedures conformed to EU Directive 2010/63EU and national regulations (RD 53/2013). All animal procedures were approved by the HGUGM Animal Experimentation Ethics Committee, the local Ethics Committees, and the Animal Protection Board of the Comunidad Autónoma de Madrid (PROEX 303.4/22).

## Results

### Printability of the 3DPS design

**Fig. 2:**
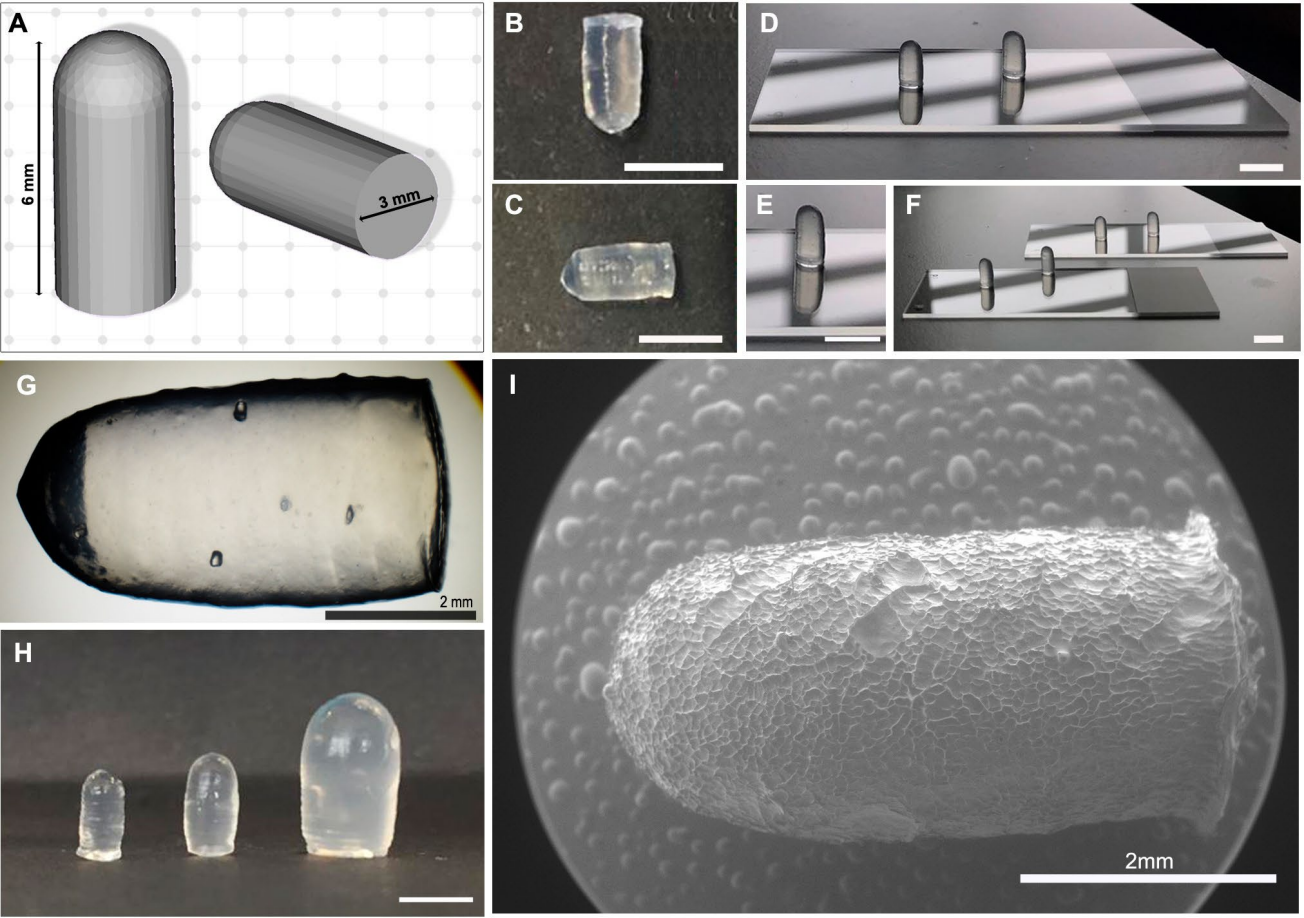
**Design characteristics of 3DPS.** **A** Auto-CAD digital design of 3DPS **B**–**C** Vertical and horizontal photographs of crosslinked 3D-3DPS, filament deposition layers remain visible. **D**, **E **,**F** Freshly printed non-crosslinked 3DPS photographs. **G** Magnification image (×20) of a freshly 3D printed and crosslinked 3DPS, filament printing layers visible **H** Image of 3DPS of small, medium and large sizes right to left disposal **I** Cryo-SEM image of a crosslinked 3DPS. Scale bar: 5 mm if not described in the image

In order to test the accuracy of the 3D-printing process, freshly printed and cross-linked 3DPS were analyzed. As depicted in Fig. 2, digital design of 3DPS was successfully printed (Fig. 2A–C). The formulations were adequately printed with the printing parameters selected for the extrusion (Fig. 2D, F). The stackability of the ink was optimal and the printing filament traces were observable after the printing process and crosslinking of the formulations (Fig. 1G). 3DPSs of three different dimensions were printed to check the printability of the process. All three dimensions were successfully constructed (Fig. 2H). The Cryo-SEM images of the formulations allowed us to accurately measure the actual dimensions of the 3DPSs. The surface of the formulations after cryo-SEM was observed to be wrinkled-shaped (Fig. 2I).

### 3DPS Loading strategy analysis

**Fig. 3:**
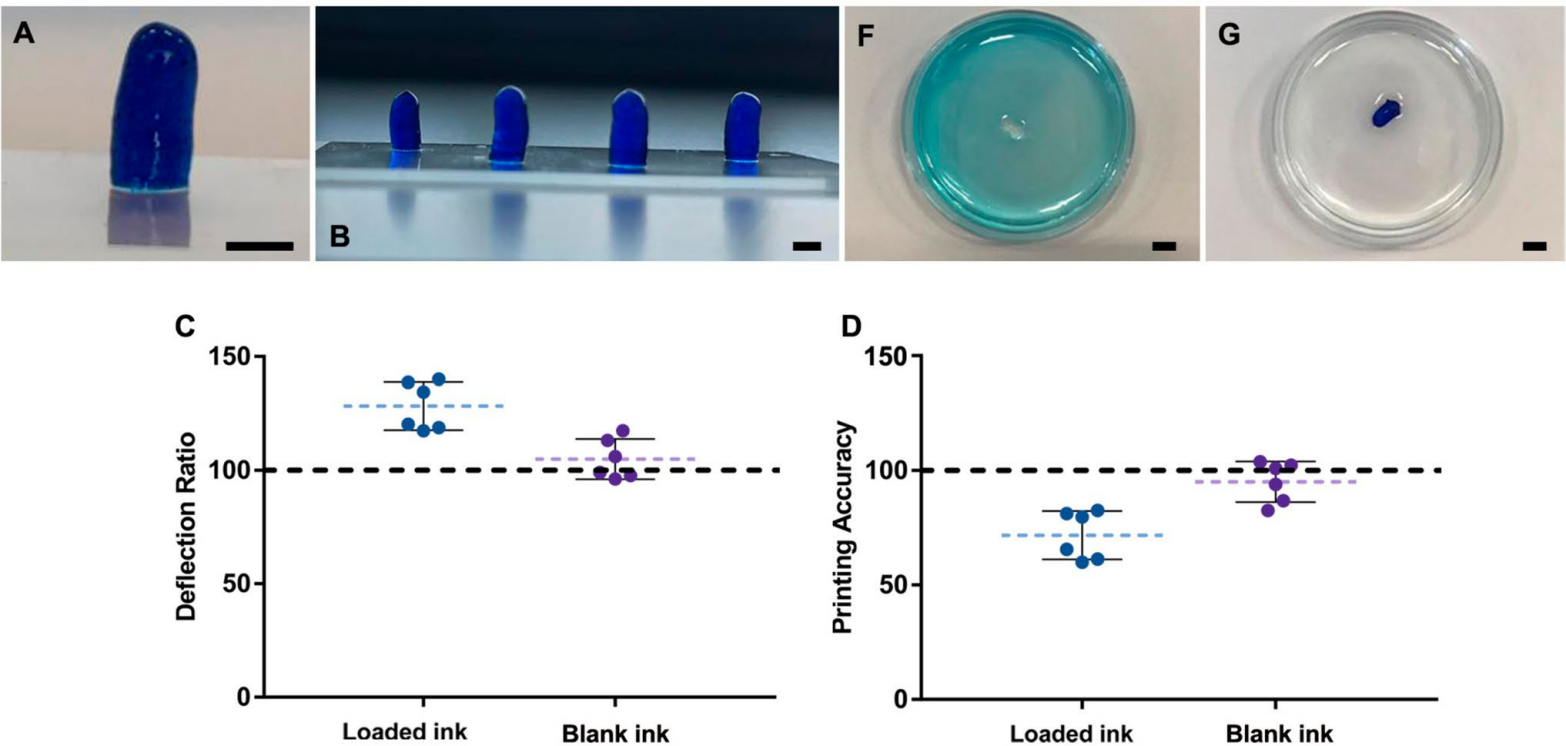
**3DPS loading strategy analysis.** **A**, **B** Methylene blue loaded ink 3DPS images. **C**, **D** Deflection ratio and Printing accuracy of loaded ink and unloaded ink 3DPS. **F**,**G** Methylene blue adsorption capacity of freshly printed and crosslinked 3DPS. Depicted 3DPS in contact with methylene blue 1% (w/v) solution (**F**) and methylene blue adsorbed inside 3DPS after 24 h (**G**)

To assess the most efficient drug-loading strategy into the 3DPS, two different drug-loading mechanisms were tested. Thus, as the most common drug loading strategy of 3D-printed devices Lap/Alg was loaded with methylene blue prior to printing as a drug model molecule. The influence of the methylene blue loading on the printability of the 3DPS was analyzed. To test the passive adsorption strategy of the 3DPSs, freshly printed and cross-linked formulations printed with non-loaded Lap/Alg ink were immersed in a methylene blue solution for 24 h to assess their adsorption capacity. The influence of each drug loading strategy on the printability of the 3DPS and the drug adsorption capacity of the 3DPS were analyzed to select the most efficient drug loading strategy for the study. The inclusion of methylene blue inside the ink prior to the printing process caused a notable deterioration in the printability of the 3DPS (Fig. 3A, B) Thus, in comparison with the loaded ink printed 3DPS (DR: 128.28 ± 10.53; PA: 71.71 ± 10.53), the blank Lap/Alg ink printed 3DPS demonstrated a better deflection ratio and printing accuracy with values closer to 100 (DR: 104.93 ± 8.83; PA: 95.063 ± 8.83) (Fig. 3C, D). Moreover, passive loading of methylene blue into the 3DPS was demonstrated to be possible with total absorption of the methylene blue into the 3DPS within 24 h of contact (Fig. 3F, G). These results identified the passive drug-loading strategy of the 3DPS as the most suitable drug-loading mechanism for the study.

### Optimization of the BSA displacement strategy

**Fig. 4:**
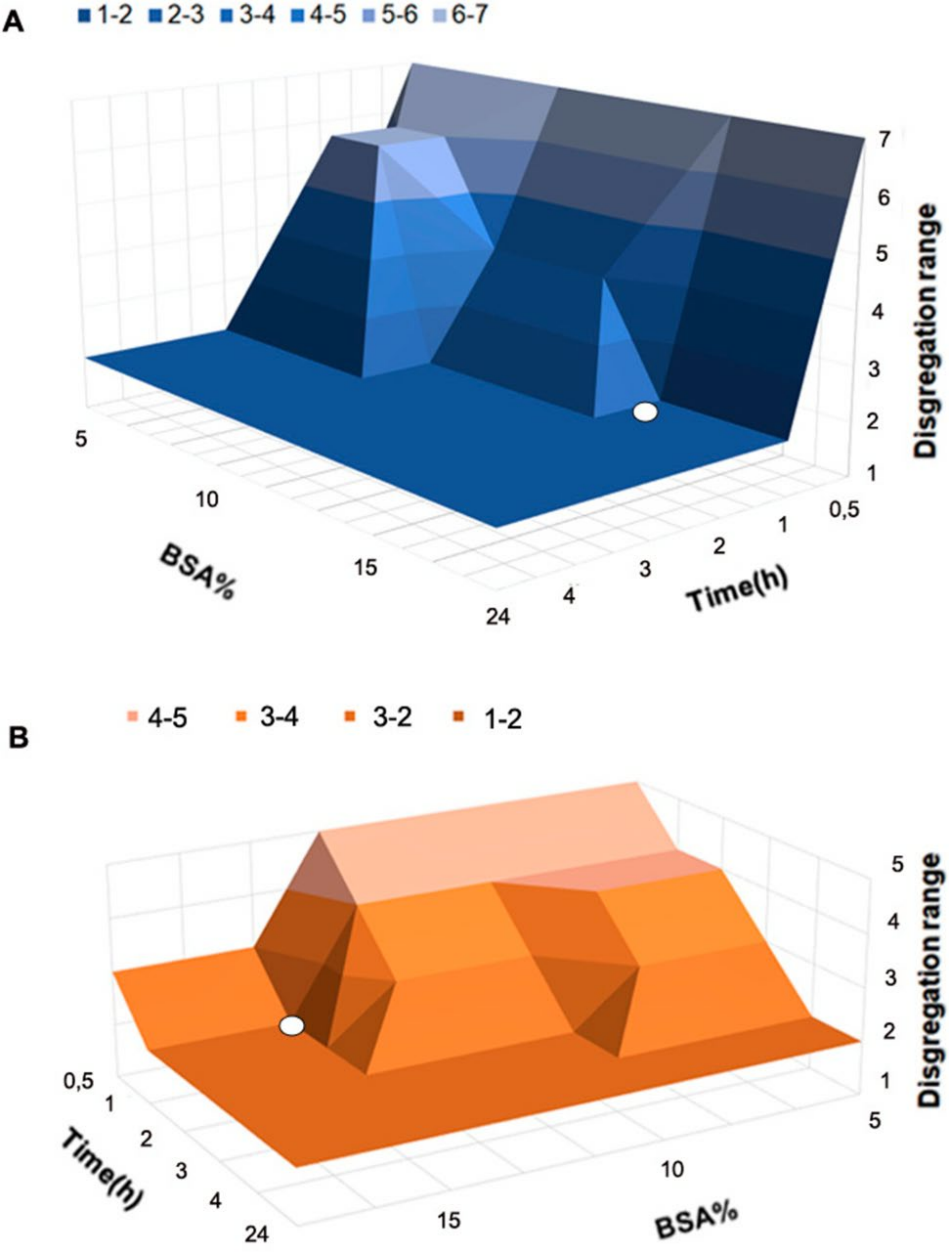
**Disintegration enhancement strategy of 3DPS with BSA displacement.** **A** Disintegration stage of displaced 3DPS over time in a USP-modified disintegration apparatus. Disintegration time ranges defined as (S1) 10–20 min, (S2) 20–40 min, (S3) 40–90 min, and (S4) ≥ 120 min. **B** Disintegration stage of displaced 3DPS over time in a 15 mL rotating tube containing 1.5 mL of PBS. Disintegration time ranges defined as (S1) ≤ 4 h, (S2) 4–6 h, (S3) 6–8 h, and (S4) ≥ 8 h

In order to develop a strategy to enhance the disintegration of the 3DPS, a BSA ionic displacement strategy was studied. This approach was undertaken in order to prove that BSA could potentially displace existing ionic interactions between the Laponite plates among the formulations, potentially leading to improved disintegration of the 3DPS. Thus, in the disintegration study, freshly printed and cross-linked 3DPS were immersed in BSA solutions of different concentrations during various exposure times prior to the study. During the study, the impact of BSA concentration and exposure time on the disintegration rate of the formulations was compared and analyzed. Therefore, we sought the most optimal displacement strategy to achieve the fastest possible disintegration of the 3DPS with the lowest BSA concentration and the shortest exposure time. As a first approach a disintegration study of the 3DPS was carried out in a USP apparatus with a high volume of dissolution media favoring the disintegration of the system. As depicted in Fig. 4A, the BSA displacement strategy proved to be effective in promoting the disintegration of the 3DPSs (Fig. 4). More accurately, the minimum exposure time to BSA to achieve the disintegration of the 3DPS in the shortest defined time interval — 10–20 min — was established as 3 h with BSA concentrations of 5 and 10% (w/v). However, an exposure time of 1 h was sufficient to obtain disintegration of the formulations in the lower range with displacement concentrations of 15% (w/v) BSA (Fig. 4A).

As a further step, the BSA displacement strategy for 3DPS was also tested under more restrictive conditions. Thus, a disintegration model in which 3DPSs were degraded in a restricted available volume and intermittent contact with the disintegration media was employed. This model aimed to more accurately mimic rectal conditions in vivo. Under these conditions, 4 h was established as the optimal disintegration time of the formulation. Thus, the minimum exposure time to achieve disintegration in 4 h was established as 3 h for concentrations of 5 and 10% BSA. (Fig. 4B). However, exposure times of 1 h to 15% (w/v) BSA solutions demonstrated to be sufficient for promoting the disintegration of the 3DPS in 4 h. Therefore, the immersion of the 3DPS with a 15% (w/v) BSA solution for 1 h was selected as the optimal and most efficient displacement strategy.

### Dextran adsorption capacity

**Fig. 5:**
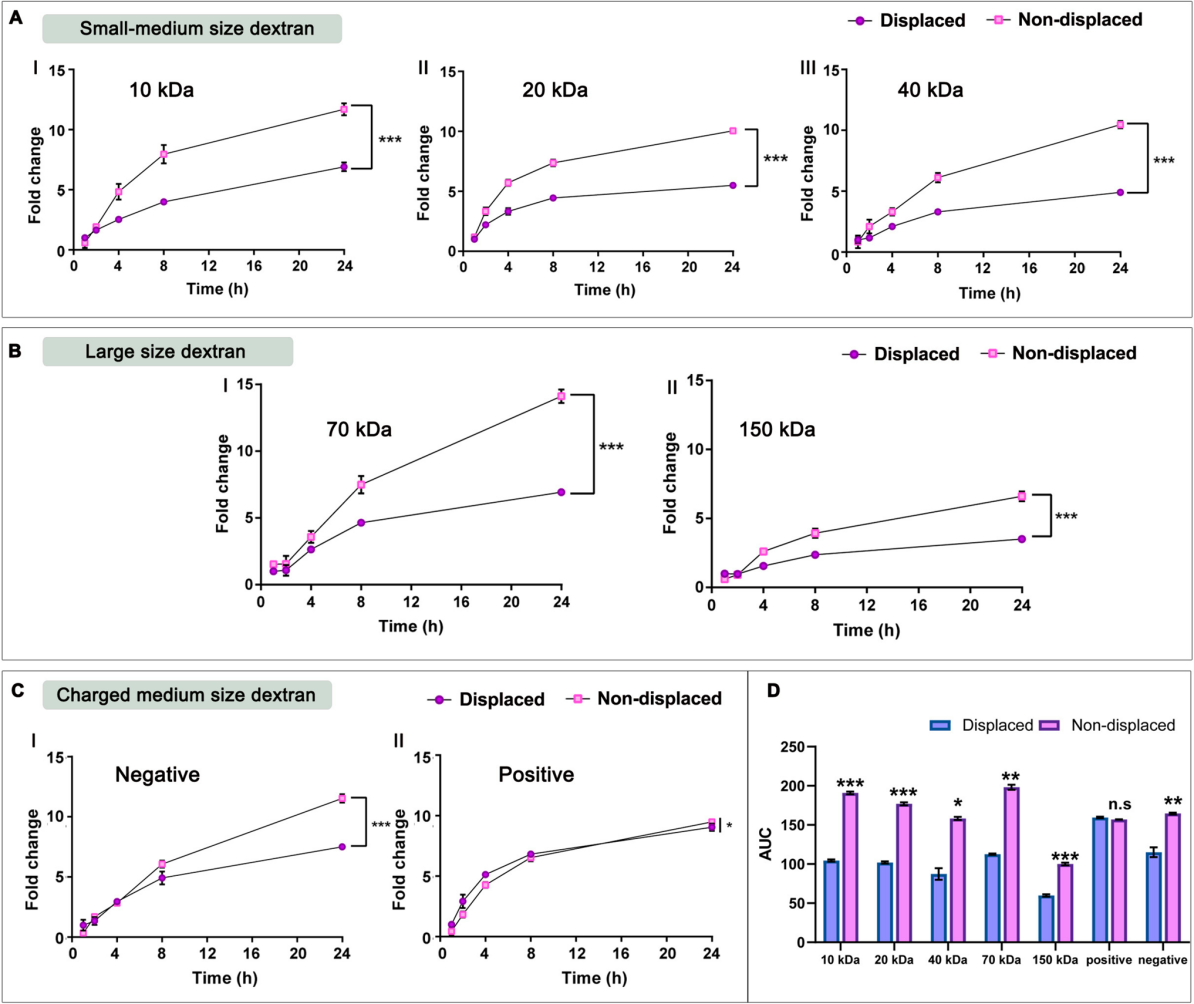
**Passive adsorption capacity of 3DPS.** **A** Adsorption capacity of small-medium size — 10, 20, 40 kDa — FITC-Dextran over time. **B** Adsorption capacity of large-size — 70, 150 kDa — FITC-Dextran over time. **C** Adsorption capacity of 40 kDa anionic and cationic FITC-Dextran over time; Normalization of fold change with respect to *t* = 1 h in the displaced group. * represents comparison of non-displaced *t*: 24 h against displaced *t*: 24 h groups: *p* < 0.05 *, *p* < 0.01 **, *p* < 0.001 ***. N.S. non-significance

The adsorption study was carried out not only to determine the adsorption capacity of molecules of different molecular weight and charge but also to elucidate the mechanism underlying this passive adsorption of the 3DPS. To this end, a comparison of the adsorption capacity of various FITC-dextran types in two distinct sets of 3DPS samples was carried out. The first set consisted of freshly printed and cross-linked 3DPS — non-displaced formulations, while the second set included 3DPS that had undertaken the BSA displacement strategy prior to the adsorption process — displaced formulations. The formulations that had undergone displacement by BSA were utilized as a control group. The purpose of this displacement by BSA was to impede the adsorption capacity of the Laponite plates through ionic interactions with the dextran molecules and shift the adsorption capacity of the formulations towards solely a passive diffusion mechanism. By doing so, we tended to assess both the loading capacity of the Laponite plates and the impact of this ionic adsorption mechanism on the adsorption of molecules with varying properties. As shown in Fig. 5, the adsorption capacity of the freshly printed and cross-linked 3DPSs demonstrated to be possible with all of the dextran types tested as a molecular model. Moreover, the adsorption capacity of the displaced formulations was lower in all types of dextran tested (Fig. 5A). The influence of the displacement strategy gave rise to minor differences in the adsorption capacity of the 3DPSs for the dextrans of higher molecular weight such as the 150 kDa dextrans (Fig. 5B).

Regarding the influence of the charge of molecules for 3DPS adsorption results, a greater affinity of positively charged dextrans was observed for the 3DPS matrix (Fig. 5C). Likewise, the displacement strategy had little effect on the adsorption of positively charged dextrans. However, negatively charged dextran adsorption had more impediments and was affected to a greater extent by the displacement strategy (Fig. 5CI/II).

These results are also observable in the AUC comparison (Fig. 5D). Thus, the influence of the displacement strategy on the adsorption of all the dextrans tested is evident, except for the negatively charged dextrans that are loaded equally in displaced and non-displaced formulations.

### Adsorption layer depth analysis

**Fig. 6:**
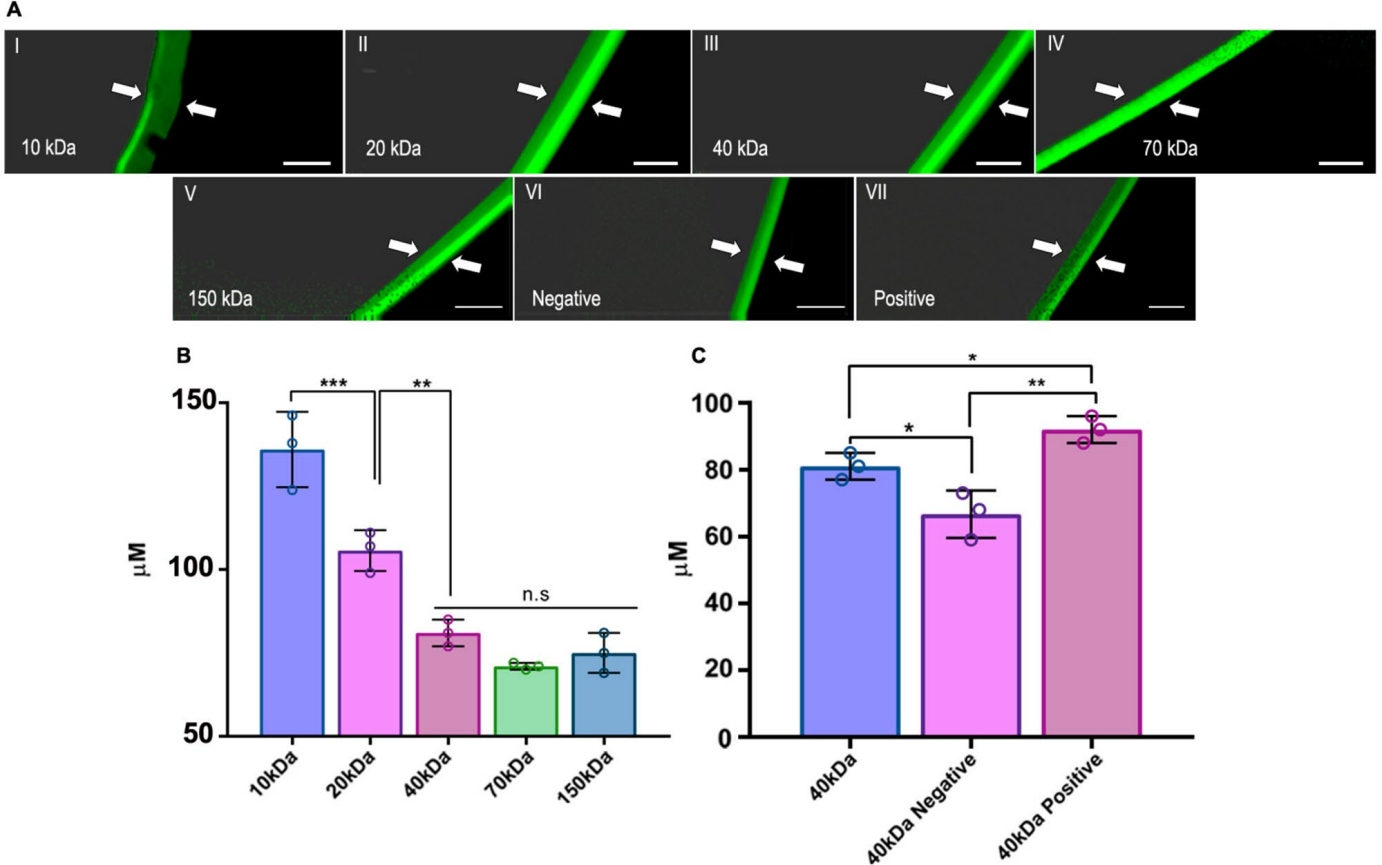
**Adsorption layer depth analysis.** **A** FITC-Dextran adsorption layer fluorescence microscopy images. Adsorption layer measured for the study, pointed with white arrows in the images. **B** Comparison of the adsorption layer depth of dextran molecules with different molecular weights. **C** Comparison of the adsorption layer depth of neutral 40 kDa, positively charged 40 kDa and negatively charged 40 kDa FITC-dextran molecules. Scale bar: 200 µm. * represents comparison against depicted groups: *p* < 0.05 *, *p* < 0.01 **, *p* < 0.001 ***. N.S. non-significance

The adsorption layer depth test was used to determine the exact location and penetration layer of the adsorbed FITC-dextrans inside the 3DPS. Moreover, this study not only served to determine the adsorption capacity of the 3DPS but also to clarify the adsorption mechanism underlying. Therefore, after completing the adsorption phase, the 3DPS samples were sectioned and examined under fluorescence microscopy. The depth of the FITC-dextran layer was quantified for each dextran type by measuring the green component in the observed sections. Firstly, the penetration layer of FITC-dextrans of different molecular weights was compared. The study revealed that the adsorbed dextrans were located in the outer cortex of the 3DPS (Fig. 6A). None of the tested dextran reached depths greater than 150 uM inside the 3DPS matrix. However, differences in layer depth were observed for the different molecular weights. The adsorption layer turned out to be significantly deeper for lower molecular weight dextrans such as 10 (135.97 µM) and 20 kDa dextrans (105.66 µM) compared to higher molecular weight dextrans such as the 70 kDa (71 µM). Furthermore, the study revealed that there were no significant differences in the adsorption layer depth between 40, 70 and 150 kDa (81 µM, 71 µM, 75 µM) dextrans (Fig. 6B).

The adsorption of molecules inside the 3DPS could be governed not only by diffusion but also by means of ionic interaction between Lap plates and loaded molecules inside its matrix. Thus, this interaction can be influenced by the charge carried by the molecules being loaded. Consequently, as a further step, we assessed the penetration capability of medium molecular weight dextrans, whose adsorption mechanism could not be solely attributed to passive diffusion and exhibited either a positive or negative charge. As a result of the study, we observed that positively charged 40 kDa dextrans revealed greater permeability (92 µM) into 3DPS and to have a larger adsorption layer depth in comparison to negatively charged 40 kDa (66.66 µM) dextrans and non-charged 40 kDa dextrans (81 µM) (Fig. 6C).

### Release capacity of 3DPS

**Fig. 7:**
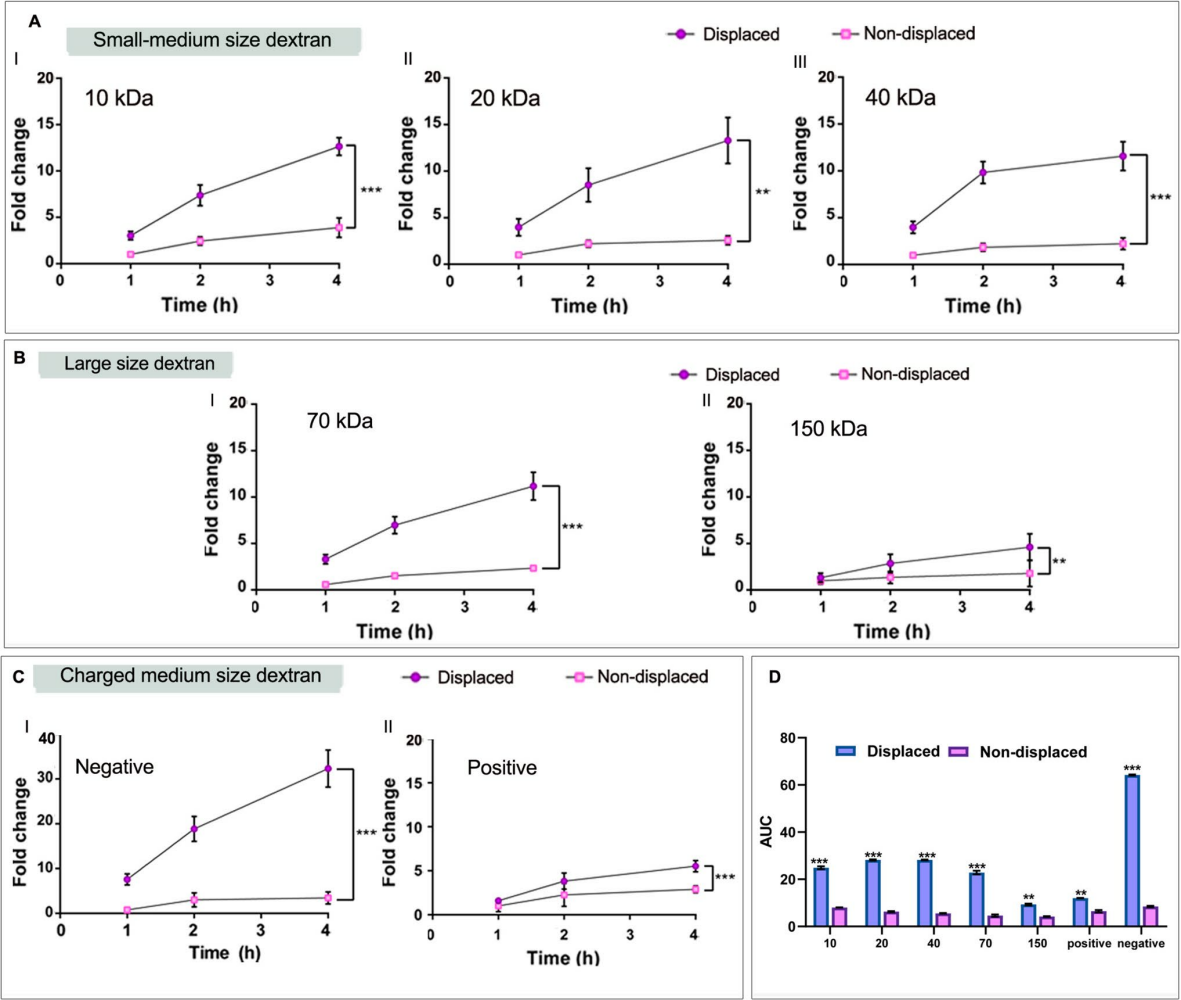
**Release capacity of displaced 3DPS.** **A** Release capacity of small-medium size — 10, 20, 40 kDa — FITC-Dextran over time. **B** Release capacity of large-size — 70, 150 kDa — FITC-Dextran over time. **C** Release capacity of 40 kDa anionic and cationic FITC-Dextran over time; Normalization of fold change with respect to *t* = 1 h in the non-displaced group. * represents comparison of non-displaced *t*: 24 h against displaced *t*: 24 h groups: *p* < 0.05 *, *p* < 0.01 **, *p* < 0.001 ***. N.S. non-significance. **D** AUC of release fold change profile graphs displayed and compared. * represents comparison of displaced against non-displaced within each dextran size groups: *p* < 0.05 *, *p* < 0.01 **, *p* < 0.001 ***

The release study was performed in order to determine the release capacity of the 3DPS as well as to test the BSA displacement strategy as a release enhancer. First, after the passive loading of the 3DPS with FITC-dextrans, the release capacity of displaced and non-displaced 3DPS was tested for different molecular weight FITC-dextran. As a result of the study, we observed that the release capacity of all of the loaded dextrans tested was demonstrated to be possible (Fig. 7). Overall, for all of the dextran types tested displaced formulation demonstrated to have higher release capacity compared to non-displaced formulations. Influence of molecular size within release capacity after displacement was proven significant. Thus, the 40 kDa dextrans were released more easily when they were displaced (Fig. 7A). The BSA displacement strategy also enhanced the release capacity of larger size dextran release such as 70 kDa dextrans (Fig. 7B/I). In contrast, the release of higher molecular weight, such as 150 kDa FITC-dextrans, proved to be less influenced by the displacement strategy, showing fewer differences in release capacity between displaced and non-displaced formulations (Fig. 7B/II).

Next, to assess BSA displacement strategy’s impact on the release of differently charged 40 kDa FITC-dextrans, we tested their release from both displaced and non-displaced 3DPS. Accordingly, the study demonstrated that the charge of the molecules shows to have an influence on the release capacity of the formulations. Thus, the negatively charged dextrans proved to be released more easily after the displacement strategy (Fig. 7C/I). Positively charged dextrans were shown to be less influenced by the displacement strategy. Thus, release capacity of positive dextrans was found to be similar between displaced and non-displaced formulations (Fig. 7C/II). The comparison data of the AUC for the release of each type of dextran confirmed that there were indeed differences in the release capacity between the displaced and non-displaced 3DPS except for the 150 kDa FITC-dextrans (Fig. 7D).

### In vivo administration and performance of 3DPS

**Fig. 8:**
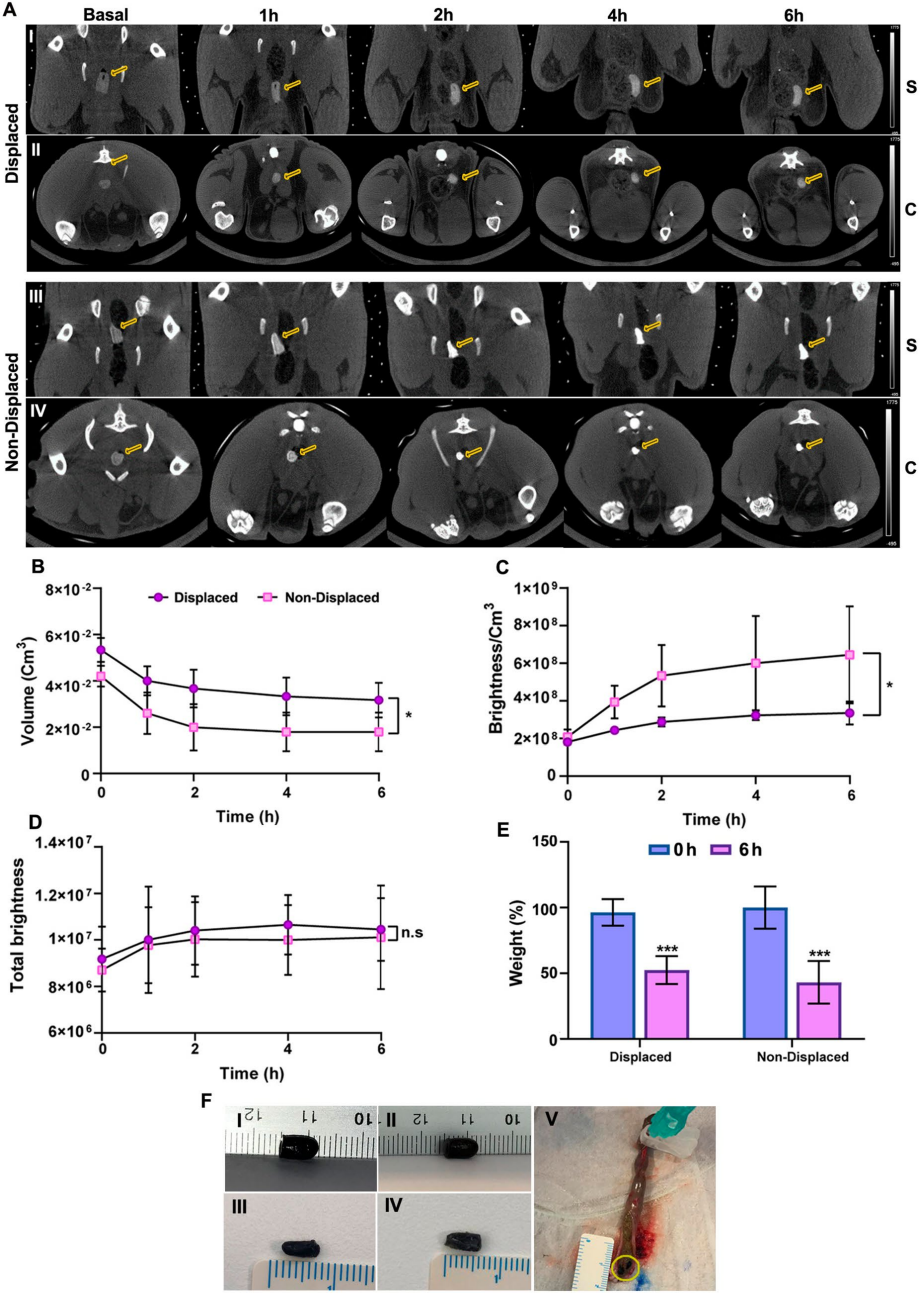
**In vivo administration of displaced and Non-displaced 3DPS.** **A** Sagittal ─S─ and Coronal ─C─ CT images of representative non-displaced and displaced 3DPS inside rat colon over time **B** ROI volume change depicted over time **C** ROI brightness normalized with ROI volume over time **D** ROI total brightness measurements over time **E** Weight loss percentage of displaced and non-displaced 3DPS over the in vivo study. **F** Photographs of displaced and non-displaced 3DPS during the in vivo study; (**I**) displaced 3DPS before the implantation and displaced 3DPS after in vivo study (**III)**. Photographs of explanted non-displaced 3DPS before the implantation (**II)** and after being explanted (**IV)**. Photograph of a rat colon explant including a 3DPS stacked into the fecal-package (**V**). * represents comparison against *t*: 0 h group: *p* < 0.05 *, *p* < 0.01 **, *p* < 0.001 ***. N.S. non-significance. (**D**) (**E**) (**F**). * represents comparison of non-displaced *t*: 6 h against displaced *t*: 6 h groups: *p* < 0.05 *, *p* < 0.01 **, *p* < 0.001 ***. N.S. non-significance

To determine the in vivo behavior of the displaced and non-displaced formulations, they were both administered in separate groups rectally in rats. For CT visualization of the 3DPS over time and also for the tracing of their disintegration in vivo, the administered formulations included a nanoparticle-based CT contrast. The ease of administration and the in vivo behavior of the formulations were examined throughout the study. The in vivo administration of 3DPS demonstrated to be possible without compromising the integrity of the formulations. Moreover, no intestinal occlusion was observed during the study. Rectal motility and transit were not negatively affected by the presence of the 3DPS. This can be corroborated by the intestinal motility observed in the advancement of feces in the CT studies. However, the presence of feces in the rectal area was significant during the study. Therefore, a progressive displacement towards the rectal area was observed in both animal groups (Fig. 8A). During the study, a progressive decrease in the volume of the 3DPSs was observed in both groups, which was slightly more noticeable in the non-displaced formulations (Fig. 8B). This decrease in volume was correlated with an increase in the brightness in the non-displaced group 3DPS, which turned out to be similar in both groups when normalizing the brightness values with the ROI volume data at each time point (Fig. 8C,D).

After the in vivo experiment, 3DPS were recovered from the rat’s colon and weighed in order to see the difference in weight loss between the displaced and non-displaced groups. As an outcome, weight loss of 3DPS was significant in both experimental groups. Both, displaced — average weight loss 51% — and non-displaced — average weight loss 49% — 3DPS demonstrated to decrease their weight similarly in comparison to their initial weight (Fig. 8E). 3DPS were easily extracted after the study and non-segmentation was observed in either of the experimental groups. Moreover, as can be observed in Fig. 8F, there was a decrease in the size of the explanted final 3DPS in both groups and formulations were located inside the accumulated fecal package (Fig. 8F, V).

## Discussion

Advances in 3D printing techniques for suppositories have been limited to the extrusion of melted filaments of lipid materials commonly used for suppository moulding [17]. Thus, little progress has been made to upgrade the novelty of the materials used for the production of 3DPS.

In our study, we employed Lap/Alg hydrogels as a novel and innovative inks for 3DPS. During our development, the Lap/Alg hydrogel proved to be well suited to the 3D printing process of the digitally designed 3DPS shapes (Fig. 2). We were able to print three different sizes of 3DPS, which demonstrated the capacity of the ink and printing technique to custom size of formulations. This was a notable advantage since hydrogel-based inks have been usually related to low printing accuracy characteristics [21]. Interestingly, the printability of the ink was notably worse when including a drug model molecule (methylene blue) inside the ink during the extrusion process. The modification of the flow characteristics of hydrogels when being loaded causes the deflection ratio and printing accuracy of hydrogels to be commonly affected and worsen [9, 31–33] (Fig. 3). Usually, 3D printing of biomedical devices for drug delivery includes the encapsulation of drugs during the printing processes; this operation method offers great limitations to the development of versatile delivery devices. Thus, the use of novel materials for 3D printing of hydrogels is often discarded due to the change in their printability when loaded with different drugs.

As the process for the technological development of an ink is complex, this slows down the development of new therapies and limits versatility of the developed inks [20, 22]. Moreover, often the stability of drugs can be altered during 3D-printing processes due to high pressure conditions inside printing cartridges or temperature increase involved in 3D printing processes. This also limits the versatility of the drug suitable for printing process inside the inks and limits the possibilities for drug encapsulation inside 3D printed biomedical devices. For this reason, a paradigm shift is necessary in which versatile inks can be loaded with different drugs. In our research, we demonstrated that the obtained 3DPS could be loaded after the printing process by means of a passive molecule adsorption mechanism (Fig. 5). This novel loading mechanism allowed us to encapsulate drug model molecules inside 3DPS without exerting a negative impact on the printability and reproducibility of the 3D printing process for the formulation production as well as without causing damage to encapsulated molecules during printing process. However, this approach would not be possible without the composition of the Lap/Alg hydrogel. The high adsorption capacity of the nanoclay allows high adsorption of drugs of different nature, giving rise to a hydrogel with high and versatile loading [34, 35]. The high drug adsorption capacity of nanoclays makes them compelling for drug delivery. Accordingly, nanoclays like Kaolinite or halloysite have been extensively employed in various studies to achieve sustained drug release [36–38]. In recent years, Lap inclusion in drug delivery systems has surged, often at low concentrations and combined with polymers like carboxymethylcellulose, hyaluronic acid, or alginate [25, 27, 39]. However, Lap is not commonly utilized as the primary excipient in formulations [9]. However, its mechanical characteristics makes Lap emerge as a valuable tool in 3D printing. This way, the use of formulations primarily composed of Lap in 3D printing offers the opportunity to harness the drug adhesion capability and rheological characteristics of the nanoclay.

Interestingly, in our study, we not only demonstrated the high adsorption capacity of the 3DPS but also designed the adsorption experiments with the objective of elucidating the adsorption mechanism responsible for such high adsorption capacity of the formulations. Lately, the high adsorption capacity of drugs on the surface of Laponite has been mostly attributed to Lap-drug ionic interactions [9, 40]. However, taking into account the charge distribution within Lap plates, the ionic interaction for drug adsorption could vary depending on the charge of the drugs. Briefly, positively charged drugs might be adsorbed within the negatively charged sides of the Lap plates, whereas negatively charged drugs might be more easily adsorbed into positively charged rims of the Lap plates. Similarly, drugs with different sizes might be adsorbed different into the Lap matrix depending on the porosity of the matrix. By employing a BSA displaced 3DPS control group during our adsorption study, we disrupted the Lap-drug interactions and this allowed us to analyze the underlying mechanism of each drug type adsorption inside 3DPS matrix.

Thus, the BSA displacement strategy proved to have great influence on the adsorption of negatively charged molecules. The negatively charged dextrans reduced their adsorption inside the displaced 3DPSs (Fig. 5CI). This occurs because the BSA molecules interact with the positively charged rims of Lap and prevent negatively charged external molecules from interacting with the matrix ionically. Probably, these positively charged rims are easily prevented by BSA since the majority of its surface is involved in the Lap-Lap or Lap/Alg ionic interactions responsible for maintaining the three-dimensional structure of the hydrogel matrix.

In the case of positively charged molecules, it was observed that the displacement strategy with BSA was not so influential on the adsorption capacity. Thus, positively charged molecules were less influenced by the interaction with BSA (Fig. 5CII). This is easily explained, since the negatively charged Lap plates, responsible for the ionic interaction with the positively charged dextran molecules, occupy a large surface area on the Lap plates which makes them more easily accessible to external molecules. Furthermore, although the BSA attached to the rims of Lap represents a steric impediment for the adsorption of positive molecules, their affinity for the Lap plates is greater, which limits the effect of BSA on the displacement of the bond.

**Fig. 9:**
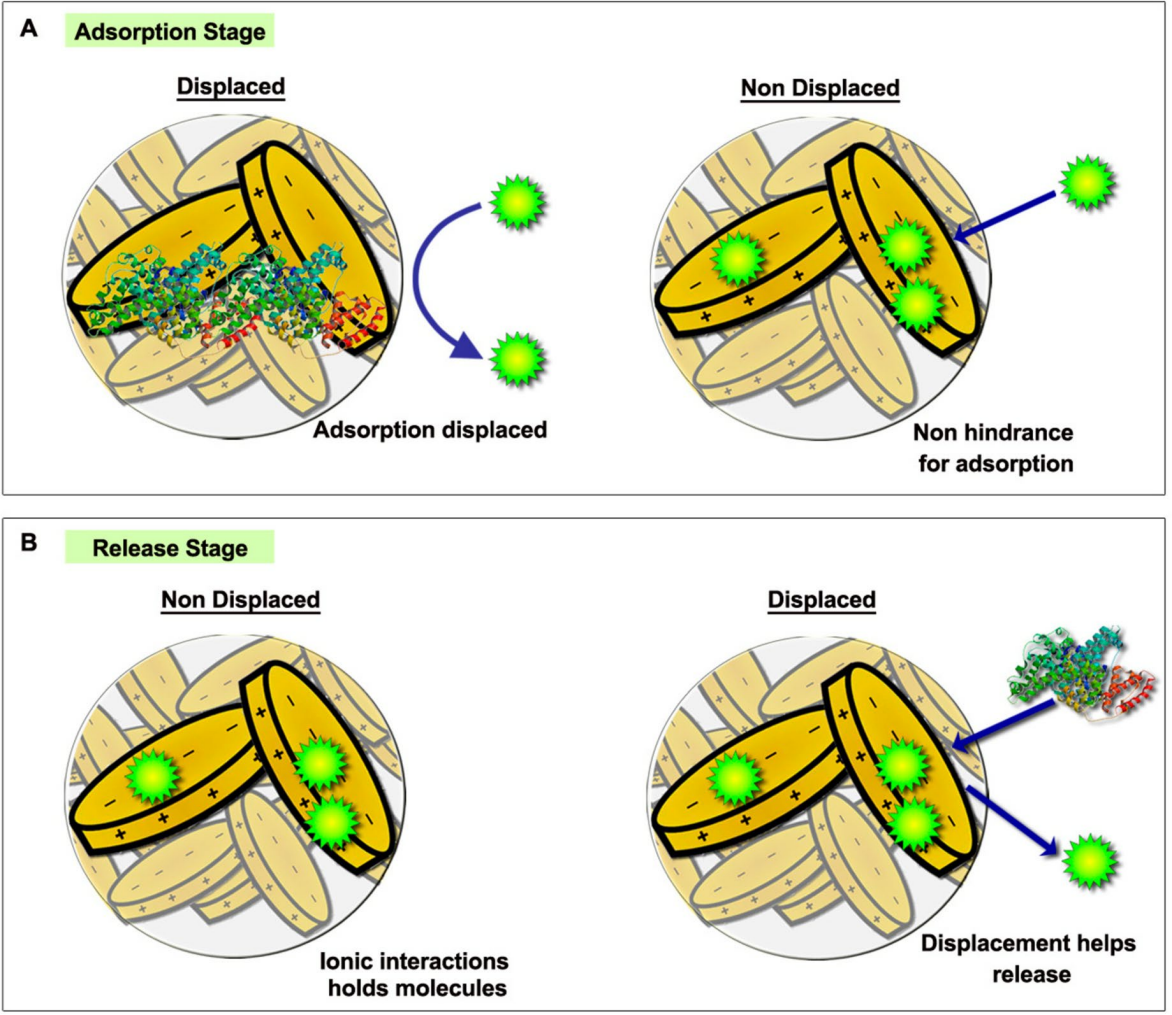
**Influence of BSA displacement strategy into adsorption and release stages of 3DPS.** **A** BSA blocks molecule binding inside Lap platelets during adsorption stage with the adsorption being favored in non-displaced matrices. **B** During release stage BSA displaces bound molecules inside Lap platelets matrix favoring molecule release within displaced 3DPS

Interestingly, the displacement strategy also affected the adsorption of uncharged dextrans of different molecular weights. Thus, the adsorption of all sizes of dextran was reduced on the displaced 3DPS. Probably, the BSA bound to the rims of Lap represents a significant steric impediment for the penetration of dextrans into the matrix. However, it is likely that the interactions between the Lap plates generate voids in which access is limited by the BSA, thus, larger molecules are prevented to a greater extent by displacement (Fig. 5A, B, D). Interestingly, these voids formed by the interactions of the Lap plates are easily accessible by the positively charged dextrans, probably because their affinity for the negatively charged areas of the Lap is greater and, therefore, they manage to displace the steric impediment that BSA represents for their adsorption (Fig. 9A).

Noteworthy, the adsorption of all of dextran types was performed within the outer cortex of the 3DPS (Fig. 6). It is likely that diffusion to more internal layers of the matrix is sterically impeded by the porosity of the hydrogel. Even so, differences in adsorption layer depth were observed with different size and charge dextran types. Once again, adsorption layer demonstrated to be deeper for positively charged dextrans, demonstrating the affinity of positively charged molecules with the 3DPS. These results indicate that the prevalent adsorption mechanism in 3DPS is not a mere passive diffusion but rather shows that the ionic interaction capacity of the molecules is a determining factor in their adsorption.

When creating a 3D printed biomedical device, it’s crucial that it not only holds the drug but also effectively releases it. However, Lap is not commonly chosen for short drug release durations because the robust three-dimensional assembly of Lap plates hinders disassembly and limits drug diffusion [26, 27]. To enhance the use of Lap in drug delivery devices, strategies are needed to facilitate Lap structure disassembly and promote drug release from the nanoclay matrices. In our study, a BSA displacement strategy was used to promote the degradation of 3DPS. As shown in Fig. 4A, B, the exposure of the 3DPS to BSA 15% (w/v) for 1 h was sufficient for enhancing 3DPS disentrapment. This increase in Lap degradation was associated with an ionic displacement mechanism caused by the intercalation of BSA molecules between the nanoclay plates. This way, the electrostatic interactions between rims and rims of Lap platelets are hindered and displaced with BSA molecules. The disassembly of these three-dimensional Lap structures allows a better wash away diffusion of the calcium ions used for the crosslinking of alginate chains, responsible for maintaining stable structural integrity [41]. Consequently, BSA boosts the disintegration of the 3DPS in contact with the aqueous medium.

Similarly, BSA displacement strategy of dextran-loaded 3DPS demonstrated to enhance their release capacity. As shown in Fig. 7, the release of displaced 3DPS was significantly higher compared to non-displaced 3DPS release capacity. This enhancement was shown to occur for all of the molecular weights tested. Interestingly, negatively charged molecules exhibited higher release enhancement when displaced. It is likely that this was due to a competitive ionic exchange between the BSA and the negatively charged molecules attached to the rims — positively charged — of the Lap plates (Fig. 9B). Thus, when being displaced by the BSA, the negative dextrans would be more easily displaced from the system. However, this displacement effect proved to have less impact for positively charged molecules as these were released similarly when displaced with BSA. Thus, the ionic displacement of BSA did not alter the release capacity of the plates — negatively charged — of the nanoplates.

As leverage, this displacement strategy significantly alters how we control the release of nanoclay, making it easier to manage and control how this versatile material is released.

A key consideration in developing new 3DPSs is their in vivo compatibility. Thus, they must be easy to handle and administer and being non-obstructive [42, 43]. The advantages of using hydrogels for rectal treatment of pathologies have been previously explored in research [44–46]. Accordingly, it has been demonstrated that the elevated swelling, high water content, and mucoadhesive properties of hydrogels provide significant advantages for rectal administration, increasing the permanence of formulations and offering low irritability and high rectal mucous layer compatibility [43]. However, the weak mechanical properties of hydrogels have been discussed often making them not appealing for rectal administration [47]. Interestingly, the 3DPS proved to comply safety characteristics when administered into the colon of rats. Both, displaced and non-displaced formulations were easily administered at the desired site, proved to be easily handled and not to break during administration. Suitably, the formulations proved to be non-obstructive, respecting intestinal transit and motility over time.

However, the anal sealing model used offered some limitations for the correct evaluation of the 3DPS in vivo. Despite fasting, the animals showed a large fecal content inside the colon (Fig. 8FV). This excessive fecal content pushed the formulations towards the anus provoking their accelerated shift (Fig. 8A). In addition, we speculate the compacted fecal content elicited high absorption of the rectal aqueous medium, causing the drying of the rectal fluid and the leakage of water from 3DPS. This water loss was also observed when analyzing the explanted 3DPS. Both, displaced and non-displaced 3DPS showed a significant weight loss (Fig. 8E) and a notable decrease in size (Fig. 8F). This water output was not accompanied by the output of the contrast. In fact, ROI analysis demonstrated an increase in brightness in the formulations over time, probably due to the concentration of the nanoparticle contrast inside the ROI (Fig. 8D). This negative diffusion of the contrast was attributed to the steric hindrance to the exit of the contrast through the hydrogel matrix.

Consequently the in vivo study allowed us to evaluate the behavior of the 3DPS in vivo but exerted limitations for the correct assessment. Still, the 12-h fasting in rats did not impede feces accumulation among the intestine of the animals. However, fasting times superior to 12 h, such as 24 h fasting times, have demonstrated to be inefficient for the complete emptying of the gastrointestinal tract of rats, being risky for the animal wellbeing by affecting their normal metabolism and behavior [48, 49].

Moreover, the anus sealing model increased the feces presence within the colonic site since it avoided the stool evacuation. Yet, anus sealing model is the only model that allows evaluating the evolution of a pharmaceutical form over an extended time in animals. Therefore, animal models without anal sealing rely the 3DPS permanence in the retention capacity of formulations within animals that do not exert voluntary retention [50]. As a conclusion, we believe there is still a challenge into obtaining animal models that allow the better in vivo evaluation of 3DPS.

## Conclusions

In our study, we have developed a novel 3D-printed hydrogel-based 3DPS. Remarkably, our 3DPS demonstrated the ability to load drug model molecules with diverse characteristics without the need to include them inside the ink during the printing process. This technological advancement allows the inclusion of various molecules within the system after printing, ensuring an outstanding versatility and reproducibility in the 3D printing process and allowing a single technological development of an ink to be suitable for the containment of several drug types. Furthermore, our study introduces the employment of BSA as a displacement strategy to enhance the disintegration and release capacity of the hydrogel systems. This novel approach enables greater release of adsorbed molecules from the hydrogel matrix, offering a solution to the previously observed problems of low drug release in studies involving this nanoclay. Moreover, our study demonstrates for the first time that 3D-printed 3DPS based on hydrogels are capable to maintain structural integrity during handling and administration, are non-occlusive at the rectal site and that are compatible with normal intestinal transit. These findings open up new possibilities in the design of 3D rectal therapies using hydrogels, proposing a paradigm shift in the materials and methods employed for conventional 3DPS production and ushering in a modern era in the field of solid rectal therapy.

## Data Availability

The data that support the findings of this study are available upon reasonable request from the authors. The authors are willing to provide the supporting data for the study upon a reasonable request.
